# Coping with COVID-19 using traditional medicine: perspectives from Joe Morolong, Northern Cape

**DOI:** 10.4102/hsag.v30i0.2773

**Published:** 2025-01-23

**Authors:** Masego M. Motsumi, Livhuwani D. Nemakonde

**Affiliations:** 1 African Centre for Disaster Studies, Unit for Environmental Science and Management, Faculty of Natural and Agricultural Sciences, North-West University, Potchefstroom, South Africa

**Keywords:** indigenous knowledge, disaster risk reduction, traditional medicine, COVID-19 pandemic, health ailments, western knowledge systems

## Abstract

**Background:**

Indigenous Knowledge Systems (IKS) have long been central to African communities, providing critical solutions to health and disaster challenges. Traditional medicine, a significant component of IKS, continues to play a vital role in addressing health needs, especially in rural areas.

**Aim:**

This study sought to gain insights on the use of traditional medicine to address the COVID-19 pandemic in five rural villages in the Joe Morolong Local Municipality, Northern Cape, South Africa.

**Setting:**

The study was conducted in Joe Morolong Local Municipality, Northern Cape, South Africa.

**Methods:**

By adopting a qualitative research design with a phenomenological approach, the study investigated participants’ lived experiences. Focus group discussions were conducted with 100 participants, conveniently sampled based on availability. Data were analysed using thematic analysis, uncovering key themes and patterns..

**Results:**

Findings revealed that most participants and their households relied on traditional medicine to treat COVID-19 symptoms. This study identified seven key medicinal plants commonly used in the community and their detailed preparation and administration methods. The findings demonstrate the essential role of traditional medicine in fostering community resilience during the pandemic, complementing conventional healthcare services.

**Conclusion:**

This study underscores the potential of IKS, particularly traditional medicine, in addressing biological hazards such as COVID-19.

**Contribution:**

The study highlights the importance of integrating traditional practices into disaster risk reduction strategies to enhance health and resilience in vulnerable communities.

## Introduction

Indigenous people are no strangers to disaster and disease (Degawan 2020). Despite global variations in indigenous peoples’ dispossession, health, and political circumstances, rural communities have depended on traditional institutions and practices to cope with seasonal stress and recurring disasters since time immemorial (Ajayi & Mafongoya 2017; Ola 2022). As Iddrisu (2017) puts it, traditional healing has its roots in ancient times. Ancient civilisations, including Mesopotamia (2900 BC), Egypt (1500 BC), China (1100 BC), India (1000 BC), Greece (300 BC) and Rome (100 AD) have all documented the use of traditional medicine (Ola 2022). According to Iddrisu (2017), a greater proportion of people in developing societies, especially those in rural areas still depend on traditional medicine entirely. This demonstrates that healthcare systems have relied on traditional medicine for a long time and will continue to do so in the foreseeable future for many nations (Mukherjee et al. 2022).

Amentie et al. (2022) argue that traditional medicine benefits individuals who depend on products derived from native plants and animals and, therefore, is a great endowment for complementing modern healthcare systems. Arguments have been put forth that indigenous knowledge (IK) is the foundation of scientifically proven knowledge, particularly in the area of medicine (Amentie et al. 2022; Mustafa et al. 2017). For instance, Amentie et al. (2022) contend that IK has served as a springboard for medical innovation and that pharmaceutical goods rely on previous discoveries, some of which have IK origins. Mustafa et al. (2017) hold similar ideas and claim that ethno-medicinal research aids in the discovery of new therapeutic medications derived from indigenous medicinal plants (Mustafa et al. 2017). Ola (2022) believes that while the goals of Western and traditional medicine are similar, their methodologies and beliefs are not. For thorough comparisons of traditional healing practices and Western medicine practices, see Mthembu (2021).

Indigenous or traditional medicine is defined as:

[*T*]he sum total of knowledge and practices, whether explicable or not, used in diagnosing, preventing, or eliminating physical, mental, and social diseases. This knowledge or practice may rely exclusively on past experience and observation handed down orally or in writing from generation to generation. (World Health Organization [WHO] 2019:8)

Traditional medicine is further referred to as an ancient and culturally linked medical practice that existed in human communities before the introduction of modern science to health (Gumbo & Singh-Pillay 2022; Iddrisu 2017). Gumbo and Singh-Pillay (2022) state that traditional or indigenous medicine is as old as Africa and was the primary form of healthcare before the birth and establishment of Western knowledge systems. Because of high value placed on health within African societies, indigenous medicine – and the communities that rely on it – occupies a significant position in Africa (Adu-Gyamfi & Anderson 2019). This enduring tradition remains central to wellness programmes and over 80% of Africa’s population relying on indigenous medicine for their healthcare needs (Gumbo & Singh-Pillay 2022). In many rural communities, traditional medicine has long been the foundation of healthcare delivery, ensuring access to vital treatments and promoting overall well-being (Dahlberg & Trygger 2009; Iddrisu 2017). This is mainly because the services of doctors, nurses, and other healthcare personnel are mainly limited to the urban and semi-urban areas in many African countries, thus disadvantaging rural communities. Other reasons cited for the high usage of traditional medicine include affordability and accessibility (Bhuda & Khazamula 2022). In South Africa, it is estimated that over 27 million people still rely on indigenous medicine, even though most people still make use of Western healthcare (Dahlberg & Trygger 2009).

African traditional medicine differs from Western medicine in the sense that it incorporates both the social and religious elements, and it focuses on the preventative and holistic health of the society rather than just an individual, thus making it scientific, social, and religious (Adu-Gyamfi & Anderson 2019). Massey and Kirk (2015) share similar views and state that indigenous people engage in empirical science that is founded in the process of revelation, traditional teaching, and observations in real-life settings when they are involved in annual cycles of subsistence activities. This leaves indigenous people with a great deal of knowledge of flora and fauna (Burgess 1999). Thus, traditional medicine consists of practices that give healing by utilising flora and fauna, particularly different plant parts such as roots, leaves, and bark, but also animal products and some spiritual approaches (Adu-Gyamfi & Anderson 2019; Bhuda & Khazamula 2022; Mthembu 2021; Ola 2022).

With the outbreak of the coronavirus disease 2019 (COVID-19) pandemic, scientists and traditional healers alike sought to find ways to prevent the spread of and treat the disease. Complementary to the development and use of vaccines for the prevention of COVID-19, indigenous communities relied on herbal treatments. Salvilla and Faller (2021) argue that people have turned to herbal medicines to combat COVID-19 because of the time it took for the medicines and vaccines to be manufactured. More importantly, indigenous communities turned to traditional medicines because they are usually left behind with limited or no access to healthcare and medical support as a result of their location in remote areas (Zhu, Zhao & Qu 2021).

Whereas there are several publications on the use of IK to prevent and cure COVID-19 in Asia (Liana & Phanumartwiwath 2021), and other parts of the African continent (Umeta Chali et al. 2021), traditional knowledge has not been broadly published (Sukarsih, Makkarennu & Soma 2022), and information on the use of indigenous knowledge systems (IKS) during COVID-19 in South Africa is limited, if any, because of a lack of recognition of the knowledge. This article aimed to gain insights into the use of traditional medicine to cope with the COVID-19 pandemic in five rural villages in Joe Morolong local municipality, Northern Cape of South Africa. This article enriches the existing body of literature, informing policy and enhancing practice by documenting the lived experience of communities that relied on traditional medicine. In the specific context of this study, the article highlights how these communities employed such knowledge to treat COVID-19 symptoms. Following this introduction, the research context and methods are articulated. The research findings are presented, analysed, and discussed before conclusions are drawn.

## Research methods and design

### Context of the study

**FIGURE 1:**
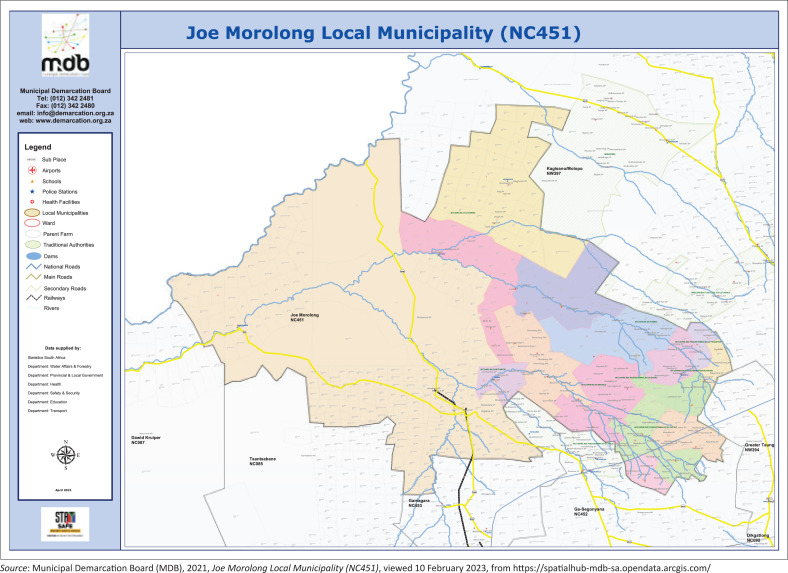
Study area: Joe Morolong Local Municipality, Northern Cape – South Africa.

This study was conducted in five rural villages of Bendell, Bothithong, Dithakong, Gasese, and Tsineng in the Joe Morolong local municipality under John Taolo Gaetsewe (JTG) District in the Northern Cape of South Africa. Joe Morolong local municipality, formerly known as Moshaweng, covers a total area of 20 172km^2^ and includes one semi-urban area, villages, and commercial farms. The local municipality is characterised by rural establishments connected mainly by gravel and dirt roads. The area is governed by tribal authorities, which include eight chiefs (*kgosis* plural = *kgosi* singular). The municipality, which has 146 villages, has been designated as the poorest in the district (Municipality 2002). The main ethnic group residing in the selected villages is the Batswana people who speak Setswana as their main home language. Therefore, Tswana cultural and linguistic heritage are predominant in the selected villages. Joe Morolong locality map is shown in Figure 1.

### Study population and sampling strategy

A qualitative research design (Maxwell 2012), specifically phenomenology was applied in this study. Phenomenology allows for capturing of lived experiences of participants (Frechette et al. 2020). Frechette et al. (2020) further state that phenomenology allows for the unearthing of phenomena from the perspective of how people interpret and attribute meaning to their existence. A convenience sampling technique based on participants’ availability at the time of the research was used to select participants from the five villages. Convenience sampling is a type of non-probability sampling where members of the target population that meet certain practical criteria such as easy accessibility, geographical proximity, availability at a given time or the willingness to participate are included for the purpose of the study (Etikan et al. 2016). This sampling technique was administered with the assistance of the chiefs of the villages, and it was deemed sufficient as the study was conducted during COVID-19 lockdown. A total of 100 community members participated in the study.

Each participant is assigned a unique code (e.g., Bend1F; Both1M), capturing the village from which they come, the focus group in which they participated in, and their gender, thus ensuring anonymity while enabling precise identification within the research context (see Table 1). This coding system facilitates the organization and analysis of interview data, allowing for clear presentation of the participants’ insights (quote verbatim).

**TABLE 1:** Participants’ codes.

Village name	FGD1			FGD2			FGD3			FGD4		
	Gender	Number	Code	Gender	Number	Code	Gender	Number	Code	Gender	Number	Code
Bendell	Female	1	Bend1F1	Female	3	Bend2F3	Male	8	Bend3M8	Female	4	Bend4F4
	Female	2	Bend1F2	Male	4	Bend2M4	Male	9	Bend3M9	Female	5	Bend4F5
	Male	1	Bend1M1	Male	5	Bend2M5	Male	10	Bend3M10	Male	13	Bend4M13
	Male	2	Bend1M2	Male	6	Bend2M6	Male	11	Bend3M11	Male	14	Bend4M14
	Male	3	Bend1M3	Male	7	Bend2M7	Male	12	Bend3M12	Male	15	Bend4M15
Bothithong	Female	6	Both1F6	Male	20	Both2M20	Female	7	Both3F7	Female	9	Both4F9
	Male	16	Both1M16	Male	21	Both2M21	Female	8	Both3F8	Female	10	Both4F10
	Male	17	Both1M17	Male	22	Both2M22	Male	25	Both3M25	Female	11	Both4F11
	Male	18	Both1M18	Male	23	Both2M23	Male	26	Both3M26	Male	28	Both4M28
	Male	19	Both1M19	Male	24	Both2M24	Male	27	Both3M27	Male	29	Both4M29
Dithakong	Male	30	Dith1M30	Male	35	Dith2M35	Female	12	Dith3F12	Female	14	Dith4F14
	Male	31	Dith1M31	Male	36	Dith2M36	Female	13	Dith3F13	Male	43	Dith4M43
	Male	32	Dith1M32	Male	37	Dith2M37	Male	40	Dith3M40	Male	44	Dith4M44
	Male	33	Dith1M33	Male	38	Dith2M38	Male	41	Dith3M41	Male	45	Dith4M45
	Male	34	Dith1M34	Male	39	Dith2M39	Male	42	Dith3M42	Male	46	Dith4M46
Gasese	Female	15	Gase1F15	Female	16	Gase2F16	Male	54	Gase3M54	Female	18	Gase4F18
	Male	47	Gase1M47	Female	17	Gase2F17	Male	55	Gase3M55	Female	19	Gase4F19
	Male	48	Gase1M48	Male	51	Gase2M51	Male	56	Gase3M56	Male	59	Gase4M59
	Male	49	Gase1M49	Male	52	Gase2M52	Male	57	Gase3M57	Male	60	Gase4M60
	Male	50	Gase1M50	Male	53	Gase2M53	Male	58	Gase3M58	Male	61	Gase4M61
Tsineng	Female	20	Tsine1F20	Female	21	Tsine2F21	Male	70	Tsine3M70	Male	75	Tsine4M75
	Male	62	Tsine1M62	Male	66	Tsine2M66	Male	71	Tsine3M71	Male	76	Tsine4M76
	Male	63	Tsine1M63	Male	67	Tsine2M67	Male	72	Tsine3M72	Male	77	Tsine4M77
	Male	64	Tsine1M64	Male	68	Tsine2M68	Male	73	Tsine3M73	Male	78	Tsine4M78
	Male	65	Tsine1M65	Male	69	Tsine2M69	Male	74	Tsine3M74	Male	79	Tsine4M79

### Data collection

The study involved 20 focus group discussions (FGDs) each guided by a structured, open-ended interview guide. In each village, four FGDs were conducted, with five participants per group. Participants included chiefs, headmen or indunas, community members, as well as chairpersons of the various village committees. Focus group discussions were chosen for their guided, interactive discussion as a means of generating rich data on complex experiences and reasons behind one’s perceptions on a particular topic (Powell & Single 1996).

### Data analysis

The collected data were analysed and presented using thematic analysis (Guest, MacQueen & Namey 2011), which is a method for identifying, analysing and interpreting patterns of meaning or themes in qualitative data (Braun & Clarke 2006). Braun and Clarke (2006) further state that a theme captures something important about the data in relation to research question and represents some level of patterned response or meaning within the data set. Therefore, thematic analysis is a good approach for finding out something about peoples’ views, opinions, knowledge, experiences or values. Specifically, a deductive approach (Bingham, 2023), also known as concept-driven coding, was utilized, where broad themes were pre-established during the design of data collection tools, guided primarily by existing literature and the researchers’ areas of interest. The analysis followed Bingham’s (2023) five phases of qualitative analysis process, which are:

- organising data;
- sorting data;
- understanding the data;
- interpreting the data;
- explaining the data.

After the first five FGDs, the researcher read through the notes and listened to the recordings to identify emerging themes or patterns after which the themes were assigned codes. This process continued until all the data were collected. Each code was given a clear definition as well as inclusion and exclusion criteria to ensure consistency in how data are categorised. The researcher then manually applied the codes to specific sections of text that fit the defined themes. This coding process was reiterative, thus reviewing and revisiting the codes periodically to identify new codes or identify existing codes that require refinement to ensure accuracy and consistency. As the patterns shaped up, the researcher examined the data patterns, relationships or differences between codes. The identified codes were then grouped into larger themes that represent the key findings of the research (see Table 2).

**TABLE 2:** Themes and sub-themes used for presenting the findings.

Theme	Sub-theme
1: Participants’ knowledge of COVID-19	-
2: Governance arrangements in the study area	2.1 Indigenous governance institutions or structures in the community 2.2 Medical institutions used during the COVID-19 pandemic
3: Medicinal plants and herbs used in the study site	-
4: IKS methods used to treat COVID-19	4.1 Preparation and use of *Artemisia afra* [Lengana] 4.2 Preparation and use of *Ruta graveolens L* (Garden Rue) 4.3 Preparation and use of *Leonotis leonurus (L.)* R.Br. (*Matekwana* or wild dagga) 4.4 Preparation and use of *Schinus molle L* (Pepper Tree) 4.5 Preparation and use of *Allium fistulosum L* [Spring Onion] and *Allium cepa* [common onion] 4.6 Mixture of African ginger, lemon and raw honey

IKS, indigenous knowledge systems; COVID-19, coronavirus disease 2019.

The themes that emerged were then used to structure the analysis and discussion of the findings.

### Ethical considerations

The ethical clearance for the study was granted by the ethics committee of the Faculty of Natural and Agricultural Science (NWU-FNAS REC) (NWU-01721-20-A9) at North-West University. Consent regarding voluntary participation, anonymity and confidentiality, and the recording of the discussion was sought and obtained verbally from participants during the data collection phase of the study.

## Results

In this section, the overall findings of the study derived from FGDs are presented.

### Socio-demographic attributes of participants

The study included 100 participants, of which 79 were males and 21 were females. Gender differences in studies on the use of traditional medicine may reveal distinct health practices, preferences or access to healthcare. For instance, men and women may rely on different remedies or perceive the effectiveness of traditional medicine differently. However, in this study, there was no distinct difference between the gender in terms of health practices, preferences or access to healthcare.

In terms of age distribution, 18 participants were between 18 years and 35 years old, 70 were between 36 years and 53 years old and 12 were over 54 years old. Age groups help identify generational differences in the use of traditional medicine. Older generations may have more experience and knowledge about traditional practices, while the younger generations may be more influenced by modern healthcare options. In this study, an overwhelming majority of participants were between the ages 36 years old and 53 years old. This age group mainly act as a bridge between older and younger generations typically responsible for the family’s healthcare decisions. This is the age group that is likely to combine the use of traditional knowledge and modern healthcare practices. This age group may have grown up learning about traditional medicine and continue to use it based on its perceived effectiveness, familiarity and accessibility.

### Participants’ knowledge of COVID-19

All participants in this study demonstrated a good knowledge and a strong understanding of COVID-19, particularly regarding the symptoms, modes of transmission, preventative measures and treatment. Across the FGDs, participants accurately identified the common symptoms of the virus, such as fever, cough and loss of taste. One person from Bothitong village remarked ‘Coronavirus is an illness of shortness of breath and coughing’ while another mentioned that ‘COVID-19 is an illness that caused coughing, inability to breathe and lack of appetite to eat’ (FGD in Gasese village). Also, participants were conversant with the mode of transmission of the virus. They pointed out that the virus is transmitted to the other person when an infected person coughs, sneezes, and talks. One participant form Dithakong FGD said ‘we also heard that the virus can come into you when you touch anything that is infected and then touch your face’. The excerpts quoted herewith capture the essence of the participants’ views to demonstrate their knowledge and understanding of the symptoms and transmission of the virus.

A significant proportion of the participants indicated that their views were informed by the information they received from public platforms, such as radio and television (TV). Others indicated that they received the information from health officials, community structures, and tribal authorities. Participants indicated that most of the information they received relates what the disease is all about, the ways it is spread, ways to prevent contracting the sickness, precautionary measures that they must take when they feel sick and ways of treating the sickness. One participant from the Bendel FGD said:

‘[*W*]e always hear on radio that the sickness is similar to influenza, but the difference is that it is more serious as it can lead to death.’ (Bend2M4)

Furthermore, most participants were able to articulate preventative measures that were advocated on radio and television to prevent the spread of the virus, particularly those that are hygiene-related; this includes cleaning hands with a soap or sanitiser, wearing of masks, avoiding handshaking and hugging, and social distancing. One participant from Bendell FGD remarked ‘everyone was wearing a mask over their nose and mouth to avoid getting the virus’.

Most participants showed knowledge regarding treatment of the virus including isolation and medical treatment. While a significant majority of participants indicated that they did not vaccinate, they were aware of the need to vaccinate so that they could not easily fall seriously ill. Those who did not vaccinate cited the remoteness of their areas, reliance on traditional medicine, and the lack of testing and vaccination facilities within their localities as some of their reasons for not vaccinating. Some participants indicated that they were reluctant to vaccinate even after vaccination services were brought to their clinics and mobile clinics were provided. However, a small number of participants who vaccinated indicated that they were ‘scared to die as there were reports of many people dying’ – FGD in Tsineng village. These participants indicated that they continued using traditional medicine even after vaccinating.

Several participants indicated that family members who tested positive were transported to the Second Eye Resort during the isolation phase, causing hardship to their families as they could not be visited. In extreme cases, such as ‘not being able to breathe and being weak’, people were referred to Kimberley Hospital for oxygen.

### Governance arrangements in the study area

In determining the governance arrangements that exist in the villages, two sub-themes emerged, and these are indigenous governance institutions or structures in the community and medical institutions used during the COVID-19 pandemic.

#### Indigenous governance institutions or structures in the community

All participants were aware of the various kinds of community structures that exist in their locality. All the villages have a mixed type of governance structure that includes the tribal authority and the political ward councillor’s system. The study found that all villages are governed by a tribal authority with the Chief as the head of the village. Participants indicated that the chiefs (Kgosi system) are firmly rooted in local traditions and customs, and the villagers still look to them for advice and guidance, enforcement of laws, and development of their villages. One participant of the Dithakong FGD said:

‘[*A*]ll chiefs make decisions on behalf of their people and are in charge of any development that must occur in the village and therefore must approve before any implementation.’ (Dith3F13)

It has been reported that during COVID-19, traditional leaders worked closely with government officials, particularly those from the Department of Health, to communicate messages on how to identify the disease and what to do when one or one’s family member is infected by the disease. Government officials leveraged on traditional leaders’ positions to relay government messages to rural communities as they are trusted and respected by communities. Thus, government officials used tribal authorities as entry points for enforcing lockdown restriction measures, and vaccination requirements, and any response and relief measures were coordinated with the chiefs.

Specifically, the participants in Bendell village were more open and enthused about their use and belief in traditional healers as an important aspect that helped them navigate the challenges imposed by COVID-19. One participant from the Bendell FGD said:

‘We prefer traditional healers; we have relied on them for year because we never had a clinic. Even after the clinic was built in Tsineng village we still visit traditional healers because the clinic is not always open.’ (Bend3M10)

Most of the traditional healers in the area, as is the case with the rest of South Africa, are members of local traditional healers’ association. Among other things, the association advocates for the recognition, welfare and rights of traditional healers within the contemporary health system; they also promote the preservation of IK or traditional medicine and its use within communities.

Whether members of the association or not, traditional healers (doctors) frequently provide spiritual and medical guidance to community members who consult them. They are experts in healing the sick and are frequently used as advisers during pandemics, such as COVID-19. For instance, they gave advice and guidance on traditional medicine to be used during COVID-19. ‘Some of us still believe in traditional healers as they are guided by our ancestors on what to do’ (Bend4F5) – Bendell FGD respondent. Another participant from the same FGD said ‘traditional healers played a critical role in supporting those infected by the disease, emotionally and spiritually’. Their roles did not end with providing emotional and spiritual support, but they were also important in recommending some of the traditional medicines to treat the virus. While most of the participants indicated that they had the knowledge of plants to treat COVID-19, some of the participants indicated that they still preferred to visit traditional healers for medication.

However, the issue of traditional healers did not go without controversy as some of the participants who do not believe in traditional healers accused them of practising witchcraft. Whereas the researchers had to guide the discussion for the issue of witchcraft not to divert the discussion, an important and even controversial accusation by those who believe in traditional healers is that those who oppose traditional leaders are the ones who visit them in secret.

Overall, both the tribal authority and the traditional healers showed their resourcefulness, influence and commitment to serve their communities during the COVID-19 pandemic. As a result of their leadership, spiritual guidance, and collaboration with government, traditional healers and traditional leaders played a crucial role in mitigating the impact of the pandemic within the communities that they serve.

#### Medical institutions used during the COVID-19 pandemic

This study found that four out of the five villages that the study participants came from had access to public health facilities, predominantly clinics. However, Gasese village lacks a clinic forcing residents to travel to Tsineng, about 10 km away on a dirt road:

‘We do not have a clinic in Gasese, so if one gets sick, we either walk or wait until we can afford to pay someone to drive us to Tsineng.’ (Gase3M56)

Because of the absence of a public transportation system in the area, residents rely on walking, donkey carts or hiring private vehicles to reach the nearest public health facility. Moreover, all villages depend on a single hospital located in Batlharos, distances ranging from 50 km from Gasese, 60 km from Tsineng, 70 km from Bendell, and about 100 km from Bothithong and Dithakong. Participants in the study expressed reluctance to visit the hospital because of poor transportation options, which are exacerbated by the poor condition of local gravel roads. One participant from Bothithong FGD said:

‘[*T*]he hospital in Batlharos is so far away and the trip is very difficult, so even when one is sick, they have to think twice before they take the journey.’ (Both3F8)

Whereas participants acknowledge the presence of local healthcare facilities, and the challenges associated with their use, many expressed frustrations that the essential services such as COVID-19 testing and vaccination were initially only available at distant health centres such as Kuruman Hospital, Tshwaragano Hospital and Kuruman Clinic. ‘People were hesitant to travel to Kuruman just to get tested or vaccinated’ (Dith2M38). This statement by one of the FGD participants captures of the decision that communities had to take for them to test for the virus or to get vaccinated. However, participants confirmed that in the later stages, testing and vaccination were extended to some of the local clinics, thus making the services more accessible by local communities. However, in spite of this, a vast majority of participants indicated that they neither tested nor vaccinated. This is so despite some participants indicating that they relied on clinics after the services were brought in, and only a few indicated that they went to medical practitioners who are in urban areas of Kuruman. The majority of FGD participants in all villages indicated that instead of visiting conventional healthcare facilities for treatment when they showed signs of cold and influenza, they relied on self-administered traditional medications while some relied on traditional healers. As indicated elsewhere in this article, participants from Bendell were not hesitant to indicate their firm belief in traditional healers as there are several in their village. Participants from other villages were reluctant to share such information on their reliance on and use of traditional healers. This was so despite the indication that traditional healers are found in every village and are easily accessible and cheap to people in need of medical care. Participants raised issues with distance to hospitals, costs of medical practitioners, and operating hours of clinics as some of the reasons why they did not visit these hospitals.

### Medicinal plants and herbs used in the study site

Most indigenous communities and rural communities’ areas including those in the study sites have a deep-rooted tradition of using indigenous medicinal plants (shrubs and herbs). Participants in the study expressed pride in using medicinal plants for the prevention and treatment of COVID-19. The statement by one of the FGD participants captures the essence of this:

‘We have always used these plants for our health but during the pandemic it felt like going back to our roots.’ (Gase1M47)

Gasese FGD Participant showed a sense of pride when discussing the use of different plants for the prevention and treatment of COVID-19. Some participants fondly related to their past experiences of their use of indigenous medicine, such as treating Lethopa, a skin disease like a boil, which they believe can be cured by a particular herb instead of being operated in hospitals:

‘We do not need to go to the hospital for Lethopa, my family has always used a special herb and it works. There is no need for a surgery.’ (Dith1M33)

As such, some participants believed that certain plants that they use for influenza (commonly known as a *flu*) and common colds have properties that can help them for the treatment of COVID-19. So, the participants found it convenient to take advantage of remedies provided by medicinal plants and herbs for the prevention and treatment of COVID-19, instead of going to the conventional healthcare facilities.

During FGDs, participants identified several medicinal plants and herbs they used to treat COVID-19 symptoms (see Table 3 for a detailed list of plants). Most of the plants – comprising of trees, shrubs, and herbs – occur naturally in the study area or are cultivated locally. Notably, many participants reported using these plants without having tested for COVID-19. One participant in the Bothitong FGD said ‘whenever I felt flu-like symptoms, I will mix some herbs, boil and drink the concoction’ (Both2M20). This is confirmed by Gumbo and Singh-Pillay (2022) who indicate that the symptoms of COVID-19 are similar to symptoms of other common colds, and therefore similar treatment and preventative measures have been adopted during the COVID-19 pandemic.

**TABLE 3:** Medicinal plants and herbs used in the study sites.

Scientific name	Family	Common/vernacular name	Botany (i.e., herb or tree)	Part used
*Artemisia afra*	Asteraceae	*Lengana* (Tswana), African wormwood (English)	Perennial, Shrub	Leaves
*Ruta graveolens L.*	Rutaceae	Benereit (Tswana), Garden Rue (English)	Shrub	Leaves
*Leonotis leonurus (L.) R.Br*.	Lamiaceae	Matekwana (Tswana), wild dagga (English)	Perennial, Shrub	Leaves
*Schinus molle L.*	Anacardiaceae	Peperboom (Afrikaans), Pepper Tree (English)	Trees	Leaves
*Siphonochilus aethiopicus* (Schweif.) B.L. Burt	Zingiberaceae	Gemere (Tswana), Wild ginger (English)	Herb	Rhizomes and roots
*Allium fistulosum L.*	Amaryllidaceae	Eiye (Tswana), Onion, Spring Onion (English)	Herb	The whole plant, bulbs, and roots
*Citrus limon*	Rutaceae	Lemone (Tswana), Lemon (English)	Trees	The fruit, juice, and peel

Note: The scientific plant names mentioned herein are confirmed by data obtained from the South African National Biodiversity Institute (SANBI) website, https://pza.sanbi.org/leonotis-leonurus.

An overwhelming majority of participants reported self-administering treatments by either boiling and drinking plants infusion or using the plants for steaming. Several participants, particularly from Bendell village mentioned visiting traditional healers after experiencing COVID-19 symptoms. However, these participants were hesitant to disclose the specific treatments of plants used citing confidentiality (ke sipiri) (Deputy Chair, Bendell FGD). This secrecy surrounding the use of indigenous medicine is what Masango (2020) argues has hindered its recognition and contributed to the perception of African traditional medicine as inferior to conventional medicine.

The scientific plant names mentioned herein are confirmed by data obtained from the South African National Biodiversity Institute (SANBI) website^1^

### Indigenous knowledge system methods used to treat COVID-19

Participants indicated that the plants listed in Table 3 were used during the COVID-19 period for treatment either individually or in combination with other herbs (concoctions). These remedies were prepared through infusion, steaming or decoction as outlined in the following sub-themes.

#### Preparation and use of Artemisia afra [Lengana]

*Artemisia afra* [lengana] emerged as the most popular remedy among participants in this study. Its widespread use is attributed to its easy accessibility, abundant availability and notable effectiveness in treating colds and influenza. The following statement by one of the participants at the Tsineng FGD is a testament to the importance of *lengana* in these communities:

‘We drank lengana. The herb was boiled and given as a tea, to those who had a cough, fever, or those struggling to breathe. Even if you were not sick, we drank it to prevent us from catching the flu.’ (Tsine2F21)

The given statement by one of the participants emphasised the importance of using *Artemisia afra* [lengana] for treating symptoms related to common cold, cough, fever, and influenza, including COVID-19. Participants noticed that the plant can be found in the wild although some prefer to cultivate it in their gardens. The preparation involved boiling the leaves with the resulting infusion administered to the patient twice daily: once in the morning and once in the evening. The following statement captures that essence of this narrative:

‘When you have influenza, take lengana, boil it, and drink in the morning and in the evening.’ (Gase2M51)

Several participants mentioned that mixing this herb with ginger accelerates the healing process. They indicated that ginger is known to boost the immune system, making it more resistant to flu viruses. Preparation methods involved boiling a few leaves in water, filtering the mixture with a clean towel and administering the decoction three times a day for a week.

Overall, this study found that *Artemisia afra* and *wynruit (Ruta graveolens)* are commonly used in the study sites, particularly as an effective remedy for influenza. The plants’ popularity surged during the COVID-19 pandemic in rural villages, highlighting its trusted medicinal value in treating respiratory infections. The findings of this study align with the research by Gumbo and Singh-Pillay (2022), conducted in KwaZulu-Natal, South Africa. Their study similarly reported the common use of *Artemisia afra* for treating colds and influenza.

#### Preparation and use of Ruta graveolens L (Garden Rue)

Participants in this study generally considered *Ruta graveolens* (Garden Rue) to be a lighter herb compared to *Artemisia afra*. Many indicated that a higher dosage of Garden Rue is required to achieve similar therapeutic effects as *Lengana*. This difference in potency influenced its use and frequency of consumption. Besides, the antiviral properties of Garden Rue likely contributed to its use by participants during the pandemic:

‘Compared with Lengana, garden rue is much milder, so a large amount needs to be consumed to achieve the results.’ (Both1M17)

Another participant mentioned, ‘to achieve similar results with lengana, one need to drink tea infused with Garden Rue more frequently.’ (Dith4M43)

Participants reported that they prepared the herb by boiling its leaves and drank the decoction as tea usually in the morning and evenings. Some participants mentioned that they regularly consume *Ruta graveolens* tea and believe that the plants’ therapeutic properties are infused in tea. Many viewed it as a routine supplement for overall health similar to multivitamin. The participants’ belief in the medicinal value of *Ruta graveolens* is supported by previous research. Asgarpanah and Khoshkam (2012) found that *Ruta graveolens* has traditionally been used to treat a variety of ailments including eye problems, rheumatism, dermatitis, pain relief and other inflammatory conditions. Enogieru et al. (2018) found that rutin, a compound found in the leaves of *Ruta graveolens*, possesses antiviral, anti-inflammatory and antioxidant properties, which make it effective in treating viral infections and related inflammatory ailments.

#### Preparation and use of Leonotis leonurus (L.) R.Br. (*Matekwana* or wild dagga)

A limited number of the participants from the villages reported using wild dagga as part of their traditional medicinal practices. The individuals described employing the ‘Boil and Drink a Teaspoon’ method, where wild dagga leaves are boiled in water and a single teaspoon of the decoction is consumed. This method was commonly used by those who believed in its effectiveness as a preventative measure against COVID-19 and its ability to treat flu-like symptoms. The findings of this study align with other research on the traditional use of wild dagga. Stefkov et al. (2016) observed that the traditional use of wild dagga has become a global trend because of its widespread application for various ailments.

Importantly, participants who used wild dagga issued a clear warning regarding the importance of correct dosing. They emphasised that improper use of the herb could lead to unintended effects such as feeling high – an experience similar to the psychoactive effects of cannabis, which is not the intended purpose. One participant said ‘drink the higher dose of wild dagga and you will feel high as if you have smoked the real dagga’ (Gase4F19). This concern is supported by Nsuala, Enslin and Viljoen (2015) who found that the dried leaves of dagga and its flowers can produce mild euphoric effects with the psychoactive impact comparable to cannabis though less intense.

#### Preparation and use of Schinus molle L (Pepper Tree)

Participants in the study reported two primary strategies for using the pepper tree to address health concerns particularly in the context of COVID-19. Firstly, many participants found that inhaling steam from the boiling leaves of the pepper tree provides relief from COVID-19, such as coughing, congestion and higher fever. This method is believed to clear the respiratory tract, reduce coughing and bring down fever. The leaves are also thought to possess anti-inflammatory properties and the ability to strengthen the immune system, which contributed to the belief in their efficacy for combating viral infections. Preparation involves boiling water and adding pepper tree leaves to the pot. Once the hot water is transferred to a container, the user wraps himself or herself in a comforter or plastic trap to steam, inhaling it for relief as described by one participant at the Dithakong FGD:

‘Steaming by means of covering oneself with a bag or plastic, steam with hot water that is infused with Peperboom leaves.’ (Dith1M32)

Secondly, to alleviate the discomfort of COVID-19, individuals who preferred not to steam, placed the tree leaves on their chests. This required placing leaves on the chest and then covering it with a cloth or bandage in such a way that the leaves would remain intact. Participants argued that the plant’s curative powers would be absorbed through the skin. Participants have, however, warned about taking the leaves orally as this might cause complications. These participants emphasised administering the treatment through the skin and through inhalation. The study’s findings align with previous research on the medicinal use of the pepper tree. Lim (2012) highlights that various parts of *Schinus molle* have traditionally been used to treat a wide range of conditions including bronchitis, tuberculosis, ulcers and wounds. This supports the participants’ use of the tree for respiratory conditions.

#### Preparation and use of Allium fistulosum L (Spring Onion) and Allium cepa (common onion)

A few participants reported using spring onion (*Allium fistulosum L)* and common onion (*Allium cepa*) to prevent getting COVID-19 infections, although this practice was not widespread in the villages. These participants believed that the vapours released from the chopped onions could help prevent acquiring influenza and COVID-19 symptoms. One participant from Bendell FGD said:

‘[*W*]e chop the anion and place it in a container and put it in a corner inside the house for the smell to fill the house, we believe that breathing the smell keeps the virus away.’ (Bend1M1)

This method reflects a traditional belief in the antimicrobial properties of onions despite its limited use among the wide community.

Also, a small number of participants mentioned using a mixture of onion and honey to treat cough and influenza. Preparation involves placing a piece of onion in boiled water mixed with honey, which they drank. One participant mentioned ‘We boil the onion with some honey and drink it. It calms the chest and helps in coughing – I always do this whenever I have a bad cold’ (Tsine3M72). Participants believed that this mixture helped to soothe the throat and chest, providing effective relief for coughing. These traditional practices are supported by scientific research. Shinwari et al. (2020) found that spring onion contains insulin-type polysaccharide with good anti-influenza activity. This adds some validity to the participants’ use of onion for treating flu-like symptoms.

#### Mixture of African ginger, lemon and raw honey

The vast majority of the FGD participants reported using a mixture of African ginger (*Siphonochilus aethiopicus*), lemon (*Citrus limon*), and honey to address the symptoms of COVID-19. They believed that this concoction known for its antibacterial and anti-inflammatory properties could treat all various forms of the virus. ‘Ginger is powerful for colds and lemons helps bring down the fever, that’s why we mix them’ (Gase1F15) said one of the Gasese FGD participants. An overwhelming majority of participants believed that honey used in this concoction must be raw, that is, unprocessed honey gathered from the veld, as it is considered superior in effectiveness. Raw honey is free from additives, so it is valued for its natural properties, which makes it the preferred choice for the participants. ‘Raw honey from the wild is the best, no preservatives, just pure. But we also bought honey from the local shops but it’s not the same’ (Dith3M41) said a participant at Dithakong FGD.

Preparation of another mixture involves boiling a piece of ginger in water, adding a slice of lemon and stirring in a tablespoon of honey. Participants emphasised that the mixture must be consumed while still warm to be most effective in relieving COVID-19 symptoms. One participant at Bothithong FGD said ‘the mixture must be drank warm – otherwise it will not work.’ (Both4M28)

The participants’ view in this study converges with other research findings. Seile et al. (2022) highlight that *Siphonochilus aethiopicus* is effective for treating coughs, colds, asthma, nausea and general discomfort. Zibaee et al. (2020) observe that lemon is beneficial for reducing infectious fevers and headaches.

## Discussion

The findings of this study reveal that most respondents demonstrated a significant understanding of COVID-19, and the measures put in place by government to curb the spread of the virus. With regard to participants’ understanding of the COVID-19 pandemic, the findings are comparable to the findings of other studies (see Amentie et al. 2022; Opoku, Aboagye & Ernest 2021). In rural areas with limited access to conventional healthcare facilities and vaccines (Ola 2022), participants primarily rely on indigenous medicinal plants for the prevention and treatment of COVID-19 symptoms. The reliance on traditional remedies was not only made a necessity because of a lack of access to conventional health facilities but also shows deep-rooted cultural practices within the community. Knowledge of traditional medicine is deeply embedded in the social and cultural capital. The pride and ownership that respondents expressed in using traditional medicine underscore the importance of IKS in building social cohesion, especially during times of uncertainty.

Despite some shortcomings in their knowledge of scientific aspects of the virus, the communities’ IK methods were significant in dealing with the virus. The study found that eight plants including the highly popular Lengana [*Artemisia afra*] were predominantly used for treating COVID-19 symptoms. These plants were prepared using various methods such as steaming, decoction and infusion, which demonstrates a wealth of local knowledge. The plants and herbs used by participants in this study are traditionally known for treating health ailments such as cough, common cold, upper respiratory infections and influenza, conditions that share similar symptoms with COVID-19. Whereas the plants cited to have been used by the study participants have not been tested in clinical trials, participants reported no side effects highlighting the safety and trust placed in the remedies by the communities.

The findings of this study show convergence with other work in the literature particularly the work of Amentie et al. (2022), which revealed that indigenous populations in Benishangul Gumuz Regional State turned to traditional medicine during the pandemic. The study by Walters et al. (2021) in Guatemala found that certain indigenous medicines have been important in strengthening people’s immune system, controlling fever, and reducing respiratory congestion. Azimi et al. (2021) confirmed similarities between COVID-19, the common colds and influenza to include coughing, fever, the feeling of fatigue, and myalgia. The study by Shinwari (2020) supports the antiviral properties of the plants used in the study sites further affirming their suitability for treating COVID-19. Specifically, the study by Liu, Van der Kooy and Verpoorte (2009) confirms that *Artemisia afra* is well known and it is used to cure a variety of disorders from coughs and colds to malaria and diabetes. Mazimba (2015) also found in their study that the leaves and stem decoction of *Leonotis leonurus* have been widely used for coughs, common colds, influenza, bronchitis, asthma, and wound healing. Alam et al. (2023) indicated in their study that *Allium fistulosum* has been used in traditional Chinese medicine for the treatment of common colds, influenza, arthritis, headache, abdominal pains, constipation, sores, and dysentery. These examples, a few of many, highlight the widespread use of indigenous medicine during the pandemic and suggest that similar practices have been observed in remote communities across various regions.

The findings of this study demonstrate the indispensable role of IK in mitigating the impacts of COVID-19, particularly in rural communities with limited access to the conventional healthcare. By leveraging their practices of using traditional medicine, communities in the study sites complemented conventional health practices, adapted to new challenges and were able to build their resilience against the virus. This study highlights the need for more inclusive approaches to disaster risk preparedness that value and integrate IK ultimately enhancing community resilience on multiple fronts. Importantly, traditional medicine must be recognised officially in South Africa as part of the national healthcare system. Also, government officials must engage with local communities to understand their practices and beliefs, fostering participation in disaster risk reduction and preparedness interventions.

### Limitations of the study

While the study was important in shedding some light on the use of traditional medicine in the study sites, the authors acknowledge limitations such as the small sample size –100 participants across five villages in a municipality with 146 villages. This could limit the generalisation of the findings to larger populations or even other contexts.

### Recommendations

By recognising and integrating IK systems into public health and disaster risk reduction strategies, governments and health institutions can leverage traditional practices to improve resilience and health outcomes in rural communities. This article suggests scientific validation of the traditional plants used in the study areas: further clinical trials are required to scientifically validate the plants’ efficacy, safety, and potential antiviral properties in treating not only COVID-19 but also other viral infections with similar symptoms. In addition, the findings highlight the need for South Africa and other Southern African countries to officially recognise traditional medicine as part of the national healthcare system if they are not yet doing so. This would involve creating a policy framework that regulates and supports traditional healers and ensures quality control. Furthermore, conventional healthcare education and awareness campaigns should cover both conventional measures as well as traditional practices. This would strengthen local capacities to manage health issues while bridging the knowledge gap between the two practices. Finally, there is a need to develop a framework to formally integrate traditional medicine into modern science, particularly in areas with limited access to conventional healthcare facilities.

### Recommendations for future research

Future research should employ longitudinal studies with larger samples to produce more robust findings. Also, future studies could focus on the policy implications of incorporating traditional medicine into the national COVID-19 response and the role of traditional medicine in building community resilience.

## Conclusion

This study sought to gain insights into the use of traditional medicine to cope with the COVID-19 pandemic in five rural villages in the Joe Morolong Local Municipality in the Northern Cape of South Africa. The study found that participants in the study sites had a wealth of knowledge about traditional medicine and they used this knowledge to address the COVID-19 pandemic. Participants were able to identify seven plants that they used for the treatment of COVID-19 symptoms. Despite being often labelled as primitive or non-scientific, indigenous practices such as the use of traditional medicine significantly contribute to the well-being and resilience of rural communities in the face of disasters, particularly in Africa and other developing nations. The findings of this study underscore that traditional medicine is a vital resource for rural communities’ health and well-being, particularly in a context where conventional healthcare systems are not accessible to everyone.

## Footnotes

1 https://pza.sanbi.org/leonotis-leonurus.
